# The Art of Destruction: Optimizing Collision Energies in Quadrupole-Time of Flight (Q-TOF) Instruments for Glycopeptide-Based Glycoproteomics

**DOI:** 10.1007/s13361-015-1308-6

**Published:** 2016-01-04

**Authors:** Hannes Hinneburg, Kathrin Stavenhagen, Ulrike Schweiger-Hufnagel, Stuart Pengelley, Wolfgang Jabs, Peter H. Seeberger, Daniel Varón Silva, Manfred Wuhrer, Daniel Kolarich

**Affiliations:** Department of Biomolecular Systems, Max Planck Institute of Colloids and Interfaces, 14424 Potsdam, Germany; Institute of Chemistry and Biochemistry, Freie Universität Berlin, Arnimallee 22, 14195 Berlin, Germany; Division of BioAnalytical Chemistry, VU University Amsterdam, Amsterdam, The Netherlands; Bruker Daltonik GmbH, Bremen, Germany; Center for Proteomics and Metabolomics, Leiden University Medical Center, Leiden, The Netherlands

**Keywords:** Glycoproteomics, Immunoglobulin, Q-TOF, Collision energy stepping CID, Synthetic glycopeptides, Glycopeptide, N-glycan, O-glycan

## Abstract

In-depth site-specific investigations of protein glycosylation are the basis for understanding the biological function of glycoproteins. Mass spectrometry-based N- and O-glycopeptide analyses enable determination of the glycosylation site, site occupancy, as well as glycan varieties present on a particular site. However, the depth of information is highly dependent on the applied analytical tools, including glycopeptide fragmentation regimes and automated data analysis. Here, we used a small set of synthetic disialylated, biantennary N-glycopeptides to systematically tune Q-TOF instrument parameters towards optimal energy stepping collision induced dissociation (CID) of glycopeptides. A linear dependency of *m/z*-ratio and optimal fragmentation energy was found, showing that with increasing *m/z*-ratio, more energy is required for glycopeptide fragmentation. Based on these optimized fragmentation parameters, a method combining lower- and higher-energy CID was developed, allowing the online acquisition of glycan and peptide-specific fragments within a single tandem MS experiment. We validated this method analyzing a set of human immunoglobulins (IgA1+2, sIgA, IgG1+2, IgE, IgD, IgM) as well as bovine fetuin. These optimized fragmentation parameters also enabled software-assisted glycopeptide assignment of both N- and O-glycopeptides including information about the most abundant glycan compositions, peptide sequence and putative structures. Twenty-six out of 30 N-glycopeptides and four out of five O-glycopeptides carrying >110 different glycoforms could be identified by this optimized LC-ESI tandem MS method with minimal user input. The Q-TOF based glycopeptide analysis platform presented here opens the way to a range of different applications in glycoproteomics research as well as biopharmaceutical development and quality control.

**Electronic supplementary material:**

The online version of this article (doi:10.1007/s13361-015-1308-6) contains supplementary material, which is available to authorized users.

**Graphical Abstract:**
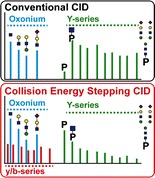
ᅟ

## Introduction

Protein glycosylation is one of the most common post-translational modifications [1]. The vast majority of membrane and secreted proteins are known or predicted to be N- and O-glycosylated [1–4]. Glycoproteins represent key molecules in many important biological processes such as cell adhesion, endocytosis, receptor activation, signal transduction, molecular trafficking, and clearance, as well as in diseases, including cancer [5, 6]. In-depth approaches to determine site-specific protein glycosylation have become indispensable tools for functional analyses of these complex biomolecules [7–9]. Specific glycans present on individual sites of a protein have been shown to be crucial for influencing the physicochemical and functional properties of their respective protein carriers. The presence or absence of a single core fucose attached to biantennary complex-type structures of the crystallizable fragment (Fc) domain of immunoglobulin G (IgG) influences the interaction of the antibody with its Fcγ-receptor, leading to a modulation of the antibody-dependent cellular cytotoxicity [10]. In IgE, the site-specific presence of an oligomannose-type N-glycan has been shown to be necessary for initiating anaphylaxis [11]. These examples illustrate that knowledge about site-specific glycosylation is an important prerequisite for studying the functional impact of protein glycosylation.

Glycoproteomic approaches, with focus on the analysis of N- and O-glycopeptides, can give detailed information on the type of structures present at a given site of a specific protein. The high sensitivity and selectivity of modern mass spectrometers in combination with different ionization methods, fragmentation techniques, and mass analyzers have made mass spectrometry the method of choice in glycoproteomics. However, analysis of glycopeptides can still be hindered by their low abundance in the entire peptide pool after proteolytic digestion because of their microheterogeneity (glycan variety attached to one glycosylation site) and macroheterogeneity (site occupancy). Furthermore, they have a general tendency to be less well ionized compared with non-glycosylated peptides [12], which can be compensated but not avoided by dedicated enrichment steps such as lectin affinity chromatography or hydrophilic interaction liquid chromatography (HILIC) [13, 14].

Glycopeptide fragmentation via tandem mass spectrometry can be achieved under standard collision induced dissociation (CID) conditions, preferentially yielding glycan fragments by cleaving the glycosidic bonds between carbohydrate units (B- and Y-ions), but rarely providing sufficient peptide b- and y-type ions (cleavage of peptide bond) for unambiguous peptide identification [15]. Higher-energy CID (HCD), in contrast, mainly results in b/y-type peptide ions as well as glycan oxonium ions and fewer Y-type ions from fragmentation of the glycosidic linkage [16]. Alternatively, electron transfer dissociation (ETD) and electron capture dissociation (ECD) keep the glycan portion on the modified amino acid mainly intact and the observed c- and z-ions produced by the N–Cα bond cleavage can provide amino acid sequence information complementary to CID fragmentation [13, 14]. However, because of their size, the majority of glycopeptides are usually detected in the *m/z* range >900 Da, thus impeding with ETD experiments. Though Alley et al. reported that ETD was able to provide useful spectra for glycopeptide precursors below *m/z* 1400 [17], our experience shows that best results are obtained for highly charged precursors (≥+3) in an *m/z* range <850 Da (personal observation).

Simultaneous acquisition of MS spectra at lower and higher collision energies (collision energy stepping CID) represents an attractive approach for selectively yielding fragment ions covering both the glycan and the peptide moieties of glycopeptides [18–21]. Nevertheless, in case higher dissociation energies are to be applied, adjusting the optimal fragmentation conditions is a prerequisite to ensure optimal fragmentation results [19], especially for the peptide part. In that context, synthetic and highly defined glycopeptide standards represent a unique and ideal tool to systematically determine the optimal conditions for glycopeptide fragmentation.

Successful large scale and software-assisted data analysis of glycopeptide product-ion spectra is not only instrument-dependent but also requires dedicated software tools. Despite the fact that great achievements have been made in this field within the last years, recent reviews on available software packages also pointed out some shortcomings due to the lack of desirable functions for comprehensive and automated glycopeptide analysis [22, 23]. Important features, such as elucidation of N- and O-glycans, matching of peptides to known protein sequences, scoring (ranking) of potential glycan and peptide moieties, parallel detection of non-glycosylated peptides, usage of product-ion data, or the option for batch inputs, have hitherto not been realized within a single software tool [22, 23].

Here we used synthetic and thus well-defined N-glycopeptides for the systematic optimization of CID energy parameters on quadrupole-time of flight (Q-TOF) instruments to obtain maximum information on both the glycan and peptide moiety within a single tandem MS experiment. Synthetic glycopeptides carrying biantennary, disialylated N-glycans were used to elucidate optimal collision energies for both the glycan and the peptide fraction. The optimized parameters were subsequently validated in a LC-ESI tandem MS online setup using tryptic peptides derived from the entire panel of human immunoglobulins (Igs) and bovine fetuin. Glycopeptide fragment data obtained from these optimized collision energy stepping CID conditions enabled software-assisted N- and O-glycopeptide data analysis in a semi-automated manner, including glycopeptide classification, compositional glycan prediction, and peptide moiety identification.

## Experimental

### Material and Methods

If not otherwise stated, all materials were purchased in high quality from Sigma-Aldrich (St. Louis, MO, USA). Trypsin (sequencing grade) was obtained from Roche Diagnostic GmbH (Mannheim, Germany). Water was used after purification with a Milli Q-8 direct system (Merck KGaA, Darmstadt, Germany). IgA (plasma), sIgA (human colostrum), IgD (plasma), IgE (myeloma plasma, lambda), IgG1 (myeloma plasma, kappa), IgG2 (myeloma plasma, kappa), and IgM (plasma) were obtained from Athens Research & Technology (Athens, GA, USA). The amino acid numbering applied for all proteins analyzed in this study is based on the respective UniProtKB entries.

### Glycopeptide Design and Purification

A tryptic N-glycosylated peptide sequence present in human protein C (entry P04070, _284_EVFVHPNYSK_293_) [24] was selected to design a small panel of synthetic N-glycopeptide standards. Besides the naturally occurring peptide sequence (termed GP-M), variations of the sequence were produced with the glycosylated amino acid either moved towards the N-terminus (GP-N) or the C-terminus (GP-C) of the peptide sequence. All glycopeptides were synthesized carrying a disialylated, biantennary N-glycan (Table 1). Glycopeptide synthesis and purification were performed as described previously [12].

**Table 1:**
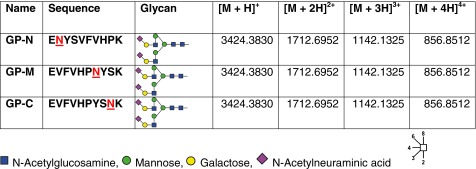
Synthetic N-Glycopeptides Used for Optimizing Q-TOF Fragmentation Conditions

### Systematic Optimization of Collision Energy Parameters for Glycopeptide Fragmentation

Synthetic glycopeptides were dissolved in 50% acetonitrile (ACN) containing 0.1% formic acid (FA) and used for direct infusion experiments (500 fmol/μL) on a Q-TOF impact II (Compass 1.9, otofControl 4.0) interfaced with an electrospray ionization (ESI) Apollo source (both Bruker, Bremen, Germany). Data was acquired using a modified version of the standard Instant Expertise method in which the product-ion spectra rate was 4–16 Hz (depending upon precursor intensity) and the number of precursors selected for fragmentation is adjusted automatically to retain a MS-tandem MS duty cycle of 3 s. For this experiment, precursors were manually selected but the tandem MS spectra rate was automatically determined as above. MS spectra were acquired at 2 Hz and precursors were isolated with a width of 3–5 Da depending on *m/z* values. Collision energies were increased from 10 to 140 eV in steps of 10 eV; for each collision energy data was acquired for 2 min in the range 150–2300  *m/z*.

### Further Optimization of Collision Energies on the Basis of LC-Separated Tryptic Glycopeptides

Correlations between collision energies and *m/z* values of glycopeptides were investigated by mixing synthetic peptides with glycopeptides derived from colon biopsies of patients with ulcerative colitis. Samples were taken with informed patient consent, ethical approval no. 39/2001. Detailed information on sample preparation can be found in the Supplementary Information.

Tryptic and synthetic peptide (500 fmol) mixtures, dissolved in 0.1% FA, were trapped on a C18 pre-column (Acclaim PepMap RSLC Nano-Trap column; 3 μm, 100 Å, 75 μm × 20 mm, Thermo Fisher Scientific, Waltham, MA, USA) and separated on a C18 analytical column (Acclaim PepMap RSLC column; 2 μm, 100 Å, 75 μm × 150 mm, Thermo Fisher Scientific) using a linear gradient from 2% buffer B (100% ACN, 0.1% FA) to 50% in 30 min, with buffer A containing 0.1% FA. The flow rate was set to 300 nL/min and oven temperature to 40 °C. The impact II was interfaced with the CaptiveSpray nanoBooster source (Bruker). MS and tandem MS data were acquired at 1 Hz from 50–3000  *m/z* with precursor selection in the range of 650–2000  *m/z*. In each MS cycle, one MS spectrum was followed by product-ion spectra of the three most intense precursors using an isolated width of 5–7.5 Da. The collision energy was set to a different value in each of 13 separate acquisitions, increasing from 10 to 130 eV in steps of 10 eV.

### Application of Optimized Fragmentation Parameters to Standard Glycoproteins

The optimized parameter settings determined for the synthetic glycopeptides were applied in the analysis of tryptic (glyco-) peptides obtained from bovine fetuin and various human Igs. Purified IgA, sIgA, IgD, IgE, IgG1, IgG2, and IgM were separated by sodium dodecyl sulfate polyacrylamide gel electrophoresis (SDS-PAGE) (Supplementary Figure 3) and the respective heavy chain bands in-gel-digested with trypsin, as described previously [25]. Fetuin was digested in solution as described elsewhere [26].

#### Setup One

Tryptic digests (1:7 dilution, dissolved in 2% ACN, 1 μL) were analyzed using the experimental setup described above with some modifications. The LC gradient was set from 5% to 50% solvent B in 60 min. Data was acquired using another modified version of the standard Instant Expertise method, which had a MS-tandem MS duty cycle of 3.5 s. MS was fixed to 2 Hz and the product-ion spectra rate was variable in the range 1.5–4 Hz depending upon precursor intensity. Precursors were automatically selected in the range of 650 to 3000  *m/z* and fragmented with an isolation width of 8–10 Da depending on *m/z* values. Collision energies were increased linearly in a *m/z* dependent manner from 55 eV at *m/z* 700 to 124 eV at *m/z* 1800, for all charge states. These values were applied to 80% of the TOF summations used for each spectrum; for the remaining 20% the collision energy was halved. In this mode of operation, ion counts from each subdivision are summed together.

#### Setup Two

Tryptic digests were also analyzed on an additional liquid chromatography-electrospray ionization-quadrupole-time of flight-mass spectrometry (LC-ESI-Q-TOF-MS) system. In detail, LC-QTOF-tandem MS analysis on a nano reverse phase (RP) column was performed on a maXis HD Q-TOF mass spectrometer equipped with a CaptiveSpray nanoBooster source (both Bruker) coupled to a Ultimate 3000 nano ultra-performance liquid chromatography system (Thermo Scientific, Breda, The Netherlands). The mass spectrometer and the LC were controlled by Hystar 3.2 (Bruker).

One microliter of the tryptic digest (1:7 dilution, dissolved in water) was loaded onto a C18 μ-pre column (PepMap100; 300 μm × 5 mm, 5 μm, 100 Å, Thermo Scientific) with 10 μL/min of 99% water/ 1% ACN/ 0.05% TFA for 5 min. (Glyco-) peptides were separated on a C18 analytical column (Acclaim PepMap RSLC; 75 μm × 15 cm, 2 μm, 100 Å, Thermo Scientific, Breda) and elution was performed at a flow rate of 700 nL/min with buffer A [water containing 0.1% FA (v/v)] and buffer B [80% acetonitrile/20% water containing 0.1% FA (v/v)]. A linear gradient of 3%–40% buffer B in 15 min was applied followed by column washing and reconditioning.

The CaptiveSpray nanoBooster was operated with acetonitrile-enriched gas (0.2 bar) and 3 L/min dry gas at 150 °C and a capillary voltage of 1200 V. MS spectra were acquired within a mass range of *m/z* 50–2800. As before, basic stepping mode was applied for the tandem MS collision energy (100%–50%) each 80% and 20% of the time, respectively, and collision energies were set as a linear curve in a *m/z* dependent manner ranging from 55 eV at *m/z* 700 to 124 eV at *m/z* 1800, for all charge states. In this setup, product-ion spectra were generated from the three most abundant precursors in a range of *m/z* 550–2800 with an isolation width of 8–10 Da depending on *m/z* values. MS was performed at a spectra rate of 1 Hz, tandem MS at 0.5 to 2 Hz dependent upon precursor intensity.

### Software Assisted Glycopeptide Data Analysis

Software-assisted glycopeptide data analysis DataAnalysis 4.4 was used for the generation of peak lists in the .XML format, which were then further analyzed using the bioinformatics platform ProteinScape 4.0 (Bruker). The classification step, which filters for glycopeptide spectra and determines the respective masses of the peptide and glycan moieties, was performed as described previously based on the presence of oxonium ions and monosaccharide distances [27].

The exact parameter settings used for the classification as well as for the peptide and glycopeptide searches are listed in Supplementary Figure 4. GlycoQuest, the glycan search engine integrated in ProteinScape, was used to search potential corresponding glycan compositions within the glycan structure database GlycomeDB (www.glycome-db.org), using the glycan masses determined in the classification step. ProteinScape 4.0 automatically replaced the experimentally determined precursor mass of respective glycopeptides with the calculated peptide mass for subsequent mascot analyses. This enabled the peptide moiety to be identified using an in-house Mascot Server version 2.3 or 2.4 (Matrix Science Ltd., UK). The collision energy optimum for peptides is the maximum of the peptide intensity coverage plotted against the applied collision energies. The peptide intensity coverage itself is a value that is the sum of the intensities of all fragments explained by peptide fragmentation divided by the sum of the intensities of all product-ion spectra signals, and thus provides a good comparative value for evaluating both presence and intensity of peptide specific fragments. The GlycoQuest score, which combines the glycan fragmentation coverage and the glycan intensity coverage, was used for determining the collision energy optimum for glycan fragmentation.

The collision energy optimum for peptides is the maximum of the peptide intensity coverage plotted against the applied collision energies. The peptide intensity coverage itself is a value that is the sum of the intensities of all fragments explained by peptide fragmentation divided by the sum of the intensities of all product-ion spectra signals, and thus provides a good comparative value for evaluating both presence and intensity of peptide specific fragments. The GlycoQuest score was used for determining the collision energy optimum for glycan fragmentation.

## Results and Discussion

### Optimization of Glycopeptide Fragmentation Using Synthetic N-Glycopeptides

A panel of defined N-glycopeptides was synthesized to systematically elucidate the optimal fragmentation conditions for glycopeptides in Q-TOF instruments. A maximum on peptide sequence as well as glycan composition data should be simultaneously acquired on the chemically distinctive fractions while minimizing dwelling and acquisition times within the instrument. The optimal settings were elucidated using the peptide intensity coverage score as an indicator for optimal peptide fragmentation conditions, whereas the GlycoQuest score indicated when a maximum of information could be obtained for the glycan moieties. This was then confirmed by manual validation of the spectra.

**Figure 1:**
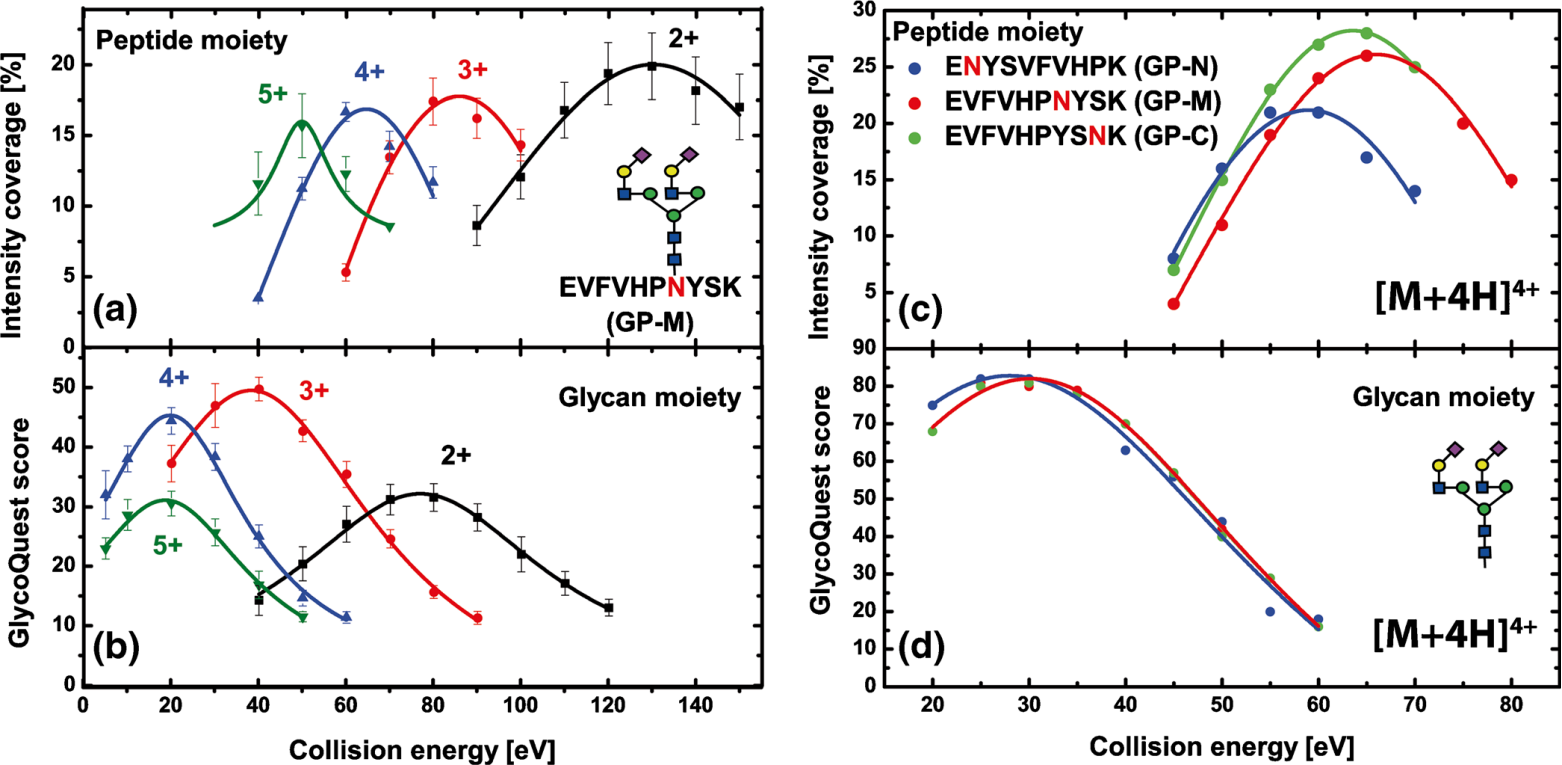
Collision energy optima for synthetic N-glycopeptides. For the peptide part, the intensity coverage was plotted versus the applied collision energy, and for the glycan part, the GlycoQuest score was used. From these plots, the respective optima were determined (refer to text for further details). For GP-M (left), the optimal collision energies were determined for charge states 2+, 3+, 4+, and 5+ **(a)** peptide part; **(b)** glycan part). Error bars represent the standard deviation determined from an average of approximately 180 individual product-ion spectra. Right: determination of the optimal collision energies for all three synthetic glycopeptides [charge 4+ **(c)** peptide part; **(d)** glycan part)]

The collision energies applied to fragment the glycopeptides were increased in 10 eV steps from 10 to 140 eV. These analyses revealed distinguished fragmentation optima for the glycan and the peptide moieties (Figure 1). Independent from the precursor charge, higher energies were required to obtain optimal dissociation of the peptide moieties. Around 30 eV provided the highest GlycoQuest score for the quadruply charged GP-M, indicating this value to be the optimal collision energy to obtain a maximum of information on the glycan moiety. For the same glycopeptide, around 65 eV were required to achieve the highest peptide intensity coverage. The data showed that the peptide moieties had a somewhat narrower fragmentation energy optimum compared with the glycan part (Figure 1a, b). We also confirmed glycopeptide fragmentation energies to be *m/z-*dependent, and lower collision energies were required for compounds with a lower *m/z* [19, 28, 29].

Interestingly, although the collision energies required for fragmentation of the peptide moiety for all three synthetic N-glycopeptides overlapped with each other, the collision energy optimum for the peptide moiety of GP-N was lower compared with the other two. In addition, the intensity coverage determined for GP-N was generally reduced over the entire collision energy range of the analyzed charge state (Figure 1c). The specific GP-N amino acid sequence as well as the position of the N-glycan present are possible explanations for the observed phenomenon, indicating that either the peptide sequence, the N-terminal proximity, or both influence the specific fragmentation behavior of this glycopeptide. In contrast, the glycan-specific collision energy optima were comparable for all three precursors, indicating that the specific amino acid sequence appears to have no or just negligible influence on the fragmentation of the N-glycans (Figure 1d). Exemplary fragment spectra for the doubly charged GP-M recorded under different collision energies (70, 100, 120 eV) are included in the Supplementary Figure 1.

### Further Optimization of Collision Energies Using LC-Separated Tryptic Glycopeptides

The collision energies and instrument settings were further optimized for a larger set of glycopeptides during a representative LC-ESI tandem MS experiment. The synthetic glycopeptide GP-M was mixed into a HILIC-enriched (glyco-)peptide fraction obtained after tryptic in-gel protein digestion of a randomly chosen SDS-PAGE band from human colon biopsy samples (Supplementary Figure 2). Overall, 16 different glycopeptide species ranging over four different charge states derived from four different proteins were successfully identified in addition to the synthetic glycopeptide GP-M (Supplementary Table 1). A range of different collision energies from 10 to 130 eV (10 eV steps) was applied to determine the optimal information output based on the GlycoQuest Score and peptide intensity coverage. The best balance was obtained when 80% of the fragmentation time was allocated to the higher energy CID conditions for acquiring peptide specific fragments. Lower energy conditions were applied for the remaining 20% fragmentation time to acquire glycan-specific fragments induced by glycosidic bond cleavages. Depending on the individual experimental requirements, more time can be allocated to the lower energy fragmentation conditions if more glyco-related fragments are desired and vice versa.

**Figure 2:**
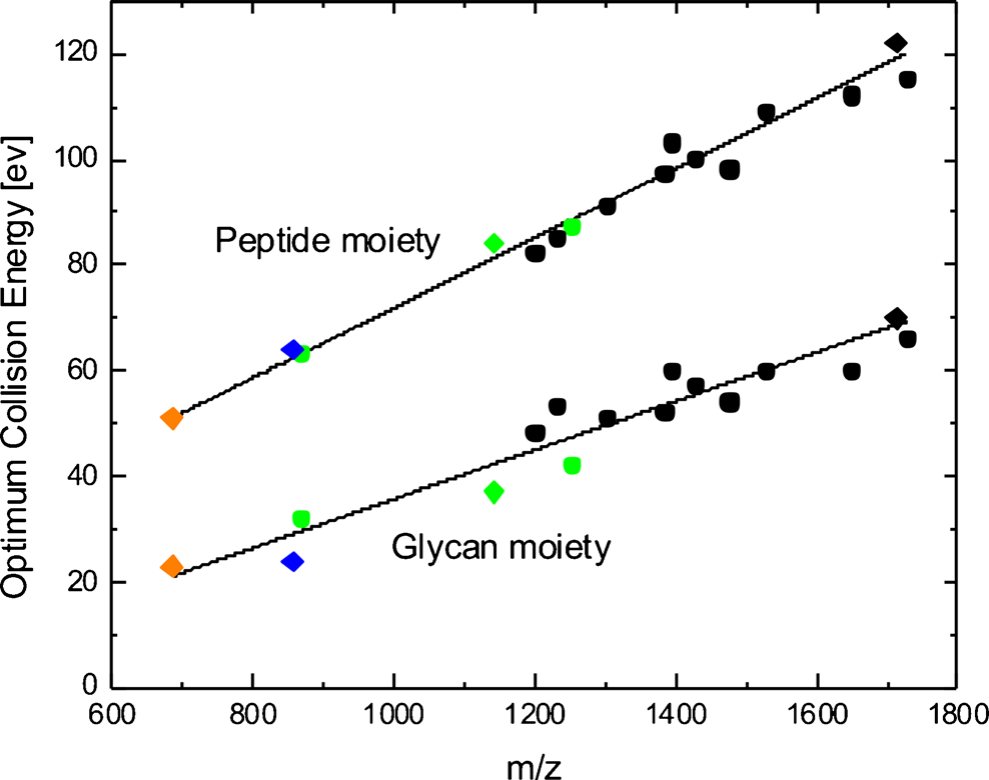
Correlation between precursor *m/z* and optimal collision energies. Synthetic glycopeptides (diamonds) were spiked into a mixture of glycopeptides (circles) enriched from a tryptic digest derived from a complex sample and analyzed via C18-RP-LC-ESI-Q-TOF tandem MS. For the [M + 5H]^5+^ species and partially for the [M + 4H]^4+^ species of the synthetic glycopeptides, values were obtained additionally by direct infusion. The optimal collision energies for peptide backbone and glycan moiety were determined based on GlycoQuest Score and peptide intensity coverage. [M + 5H]^5+^ species are indicated in orange, [M + 4H]^4+^ in blue, [M + 3H]^3+^ in green, and [M + 2H]^2+^ in black

A linear dependency of the collision energy optimum and the *m/z* ratio was observed when the collision energies were stepwise incremented. This further confirmed the general trend that precursors with higher *m/z* ratios require higher collision energies for optimal fragmentation (Figure 2). Depending on the particular peptide composition and the distribution of certain amino acids within a peptide sequence, the availability of readily mobile protons may vary, resulting in slightly deviated optimized collision energies. This is best exemplified by our data obtained for glycopeptide GP-N (Figure 1c), but has also been described previously [19, 30]. Nonetheless, the optimized collision energies initially determined on the synthetic N-glycopeptides could be further refined using glycopeptides with various peptide backbones and glycan structures. Based on these results, a collision energy method was employed using 55 eV at *m/z* 700 to 124 eV at *m/z* 1800 as high-energy values for 80% of the fragmentation time before the energy values were halved for the remaining 20%.

### Method Application: Analysis of Tryptic (Glyco-)Peptides Derived from Various Human Immunoglobulins and Bovine Fetuin

With the optimized collision energy method at hand, we set out to benchmark the method using tryptic (glyco-)peptides obtained from various human Igs (IgA1+2, sIgA [secretory component, joining chain, IgA1+2], IgD, IgE, IgG1+2, and IgM) as well as bovine fetuin. These glycoproteins contain both N- and O-glycopeptides and are well described (references in Table 2), which makes them prime model samples for a systematic evaluation of the optimized collision energy settings as well as the automated glycopeptide data analysis software embedded in ProteinScape 4.0.

**Table 2:** Overview of theoretical and automatically identified (by PS 4.0) human Igs and bovine fetuin glycosylation sites

Theoretical sites/ tryptic peptide	Detected		Peptide sequence (smallest detected or theoretical possible peptide portion indicated)	GF	Detected compositions (Hex,HexNAc,NeuAc,dHex)	Ref.
	PS4	Man				
**IgA (serum derived)**						
**IgA1 (P01876); IGHA1_HUMAN**						
N144	X		LSLHRPALEDLLLGSEANLTCTLTGLR	2	5,4,1,0 / 5,5,1,0	[40–44]
N340	X		LAGKPTHVNVS(*VVMAEVDGTCY*) (unspecific cleavage)	3	5,4,1,1 / 5,4,0,1 / 5,5,1,1	[41, 44]
O105, O111, O113, O119, O121	-	X	HYTNPSQDVTVPCPVPSTPPTPSPSTPPTPSPSCCHPR	9	3,4,1,0 / 4,4,1,0 / 3,5,1,0 4,5,1,0 / 4,5,2,0 / 4,4,2,0 3,4,2,0 / 4,4,3, 0 / 5,5,3,0	[37, 44, 45]
**IgA2 (P01877); IGHA2_HUMAN**						
N47	-	-	VFPLSLDSTPQDGNVVVACLVQGFFPQEPLSVTWSESGQNVTAR	n.d.		[46]
N92	X		HYTNPSQDVTVPCPVPPPPPCCHPR	n.d.		[36]
N131	X		LSLHRPALEDLLLGSEANLTCTLTGLR	2	5,5,1,0 / 5,4,1,0	[41, 46]
N205	X		TPLTANITK	3	5,4,1,1 / 5,5,1,1 / 5,5,0,1	[40, 41, 46–48]
N327	-	-	MAGKPTHVNVSVVMAEVDGTCY			[41, 46, 48]
**sIgA (colostrum derived)**						
**Polymeric immunoglobulin receptor (P01833); PIGR_HUMAN**						
N83, N90	-	X	ANLTNFPENGTFVVNIAQLSQDDSGR		10,8,1,4 / 10,8,0,6	[24, 37, 40, 46, 48, 49]
N35	-	X	GLSFDVSLEVSQGPGLLNDTK	2	5,4,1,2 / 5,4,0,2	[37, 46, 49]
N186	-	X	QIGLYPVLVIDSSGYVNPNYTGR	2	5,4,1,1 / 5,4,1,2	[37, 46, 48, 49]
N421	-	X	LSLLEEPGNGTFTVILNQLTSR	4	5,4,1,2 / 5,4,0,2 / 5,4,0,3 5,4,21	[24, 37, 40, 46, 48–50]
N469	X		VPGNVTAVLGETLK	2	5,4,2,0 / 5,4,1,1	[24, 37, 40, 41, 46, 48–50]
N499	X		WNNTGCQALPSQDEGPSK	3	5,4,1,1 / 5,4,0,1 / 5,4,1,0 (more manually detected)	[37, 46, 48, 49]
**Joining chain (P01591); IGJ_HUMAN**						
N71	X		ENISDPTSPLR	1	5,4,1,0	[24, 37, 40, 46, 48, 50]
**IgA1 (P01876); IGHA1_HUMAN**						
N144	X		LSLHRPALEDLLLGSEANLTCTLTGLR	7	8,2,0,0 / 7,2,0,0 / 6,2,0,0 / 5,2,0,0 / 3,5,0,0 / 5,4,0,0 / 4,5,0,0	see above
N340	X		LAGKPTHVNVSVVMAEVDGTCY	1	6,2,0,0	see above
O105, O111, O113, O119, O121		X	HVKHYTNPSQDVTVPCPVPSTPPTPSPSTPPTPSPSCCHPR	6	3,4,1,0 / 4,4,1,0 / 3,4,2,0 / 4,5,1,0 / 4,4,2,0 / 4,4,3 0 (more manually detected)	see above
**IgA2 (P01877); IGHA2_HUMAN**						
N47	X		(*VFPLSLDSTPQDGNVVVACLVQGFFPQEPL*)SVTWSESGQNVTAR (unspecific cleavage)	1	3,5,0,1	see above
N92	X		HYTNPSQDVTVPCPVPPPPPCCHPR	n.d.		see above
N131	X		LSLHRPALEDLLLGSEANLTCTLTGLR	5	8,2,0,0 / 7,2,0,0 / 6,2,0,0 / 5,2,0,0 / 3,5,0,0	see above
N205	X		TPLTANITK	6	5,5,0,1 / 5,5,1,1 / 4,5,0,1 / 4,5,1,1 / 4,4,1,1 / 3,5,0,1	see above
N327	-	-	MAGKPTHVNVSVVMAEVDGTCY	n.d.		see above
**IgG**						
**IgG1 (P01857); IGHG1_HUMAN**						
N180	X		EEQYNSTYR	7	5,4,0,1 / 5,4,1,1 / 4,5,0,1 4,4,0,1 / 3,5,0,1 / 3,4,0,1 4,4,0,0	[41, 50, 51]
**IgG2 (P01859); IGHG2_HUMAN**						
N176	X		EEQFNSTFR	5	4,4,1,1 / 3,5,0,1 / 3,4,0,1 4,5,0,1 / 4,4,0,1	[40, 41, 50]
**IgM (P01871); IGHM_HUMAN**						
N46	X		YKNNSDISSTR (1 missed cleavage)	13	6,3,1,1 / 6,3,1,0 / 6,2,0,0 5,5,0,1 / 5,5,1,1 /5,5,1,0 5,4,0,1 /5,4,2,1 / 5,4,1,1 5,4,2,0 / 5,4,1,0 / 4,3,0,1 4,3,1,1	[41, 47, 50, 52]
N209	X		GLTFQQNASSMCVPDQDTAIR	4	5,5,0,1 / 5,5,1,1 / 5,4,2,1 5,4,1,1	[41, 52]
N272, N279	-	-	THTNISESHPNATFSAVGEASICEDDWNSGER			[52, 53]
N439	X		STGKPTLYNVSLVMSDTAGTCY	1	6,2,0,0	[52, 53]
**IgE (P01854); IGHE_HUMAN**						
N21, N49	-	-	NIPSNATSVTLGCLATGYFPEPVMVTWDTGSLNGTTMTLPATTLTLSGHYATISLLTVSGAWAK	n.d.		[31, 35, 54]
N99	X		VAHTPSSTDWVDNK	3	5,5,2,1 / 5,5,1,1 / 5,5,0,1	[31, 35, 54]
N146	-	-	ILQSSCDGGGHFPPTIQLLCLVSGYTPGTINITWLEDGQVMDVDLSTASTTQEGELASTQSELTLSQK	n.d.		[31, 35]
N252	X		GTVNLTWSR	1	5,5,0,0	[31, 35, 54]
N275	X		NGTLTVTSTLPVGTR	8	9,2,0,0 / 8,2,0,0 / 7,2,0,0 6,2,0,0 / 5,2,0,0 / 5,4,0,1 /5,4,0,0 /5,3,0,0	[31, 35]
**IgD (P01880); IGHD_HUMAN**						
N225	-	X	VPTGGVEEGLLERHSNGSQSQHSR (1 missed cleavage)	3	10,2,0,0 / 9,2,0,0 / 8,2,0,0	[24, 31, 55]
N316	X		EVNTSGFAPARPPPQPGSTTFWAWSVLR ^a^	n.d.	^a^	[24, 31, 50, 55]
N367	X		TLLNASR	5	5,4,2,1 / 5,5,0,1 5,5,2,1 / 5,5,2,0 / 4,5,0,0	[31, 55]
O109, O110, O113	X		AQASSVPTAQPQAEGSLAK	1	1,2,2,0	[31, 32]
O109, O110, O113, O126, O127, O131, O132	-	X	AQASSVPTAQPQAEGSLAKATTAPATTRNTGR (2 missed cleavages)	3	5,5,6,0 / 5,5,7,0 / 5,5,5,0	[31, 32]
**Fetuin (P012763); FETUA_BOVIN**						
N99	X		RPTGEVYDIEIDTLETTCHVLDPTPLANCSVR	1	6,5,3,0	[39, 51, 56, 57]
N156	X		LCPDCPLLAPLNDSR	2	6,5,3,0 / 5,4,2,0	[39, 51, 56, 57]
N176	X		VVHAVEVALATFNAESNGSYLQLVEISR	n.d.	^b^	[39, 51, 56, 57]
O271,O280, O282,O296	-	-	VTCTLFQTQPVIPQPQPDGAEAEAPSAVPDAAGPTPSAAGPPVASVVVGPSVVAVPLPLHR	n.d.		[39, 58]
O334,O341	X		TPIVGQPSIPGGPVR	1	1,1,1,0	[39, 58]

n.d. = not detected; PS4 = glycopeptides identified by ProteinScape 4.0 GlycoQuest and Mascot; Man. = manually identified; Ref. = literature reference, glycoforms (GF) = corresponds to the number of different glycoforms detected and identified by product-ion spectra; red letters indicate potential glycosylation sites reported in literature within the peptide sequence; blue letters indicate missed cleavages by trypsin.

^a^Site N316 has been described to be modified with a carbohydrate previously [55]. A low intensity signal of the unglycosylated tryptic peptide was detected; however, due to the specific sequence and size of a peptide including a missed cleavage at R313 (followed by E314) it is highly possible that any glycosylated forms were not detected by the specific setup used in this study.

^b^This peptide was just detected in its unglycosylated form.

**Figure 3:**
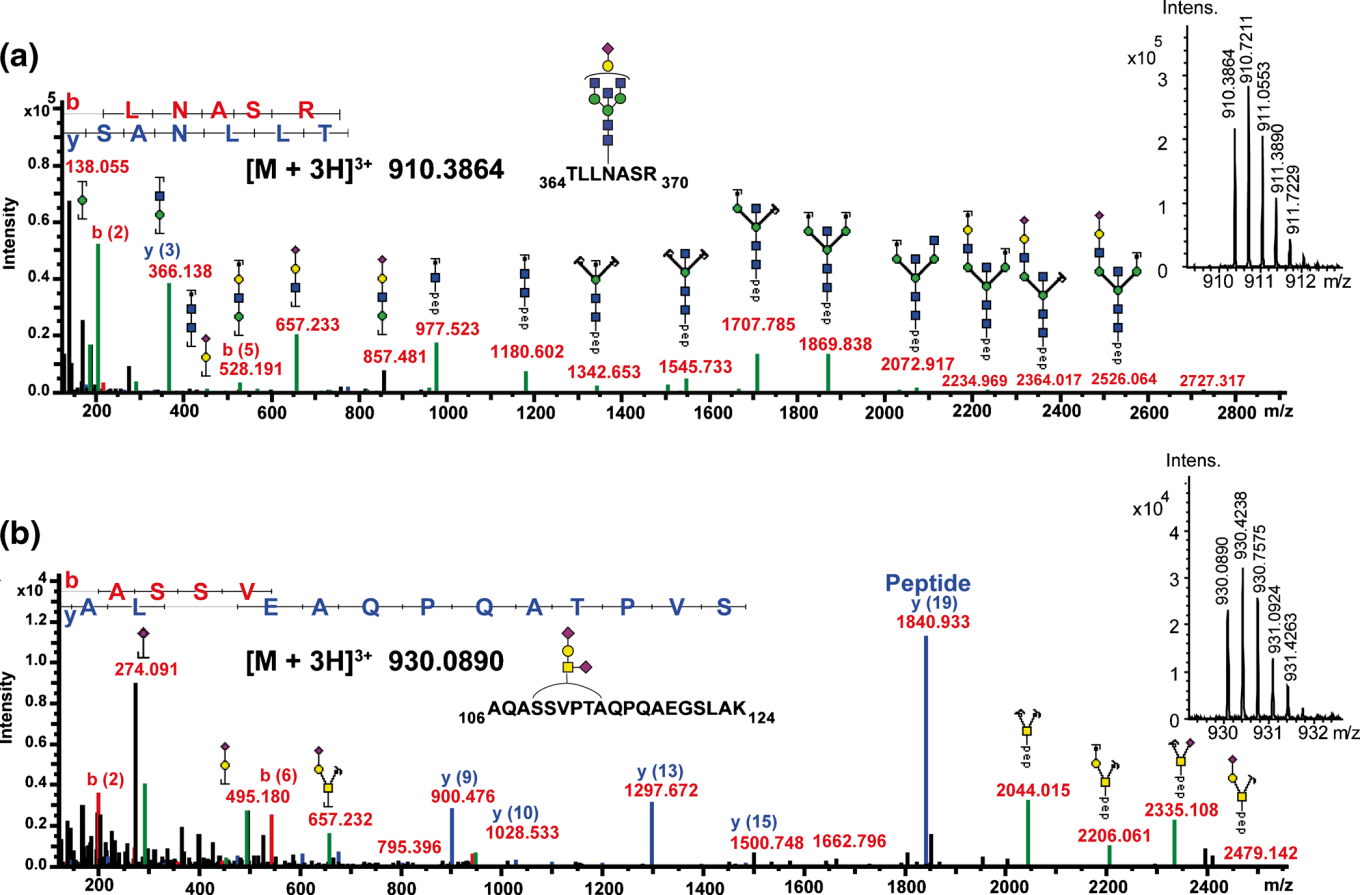
Representative spectra of automatically assigned N- and O-glycopeptides from IgD using the optimized Q-TOF collision energy stepping dissociation method. **(a)** tryptic N-glycopeptide TLLNASR (N367, [M + 3H]^3+^ at *m/z* 910.386) carrying a Hex_5_HexNAc_5_NeuAc *N*-glycan. The specific glycopeptide spectrum provided sufficient information to assign it as a monosialylated diantennary *N*-glycan with a bisecting GlcNAc. **(b)** Tryptic *O*-glycopeptide AQASSVPTAQPQAEGSLAK (Ser109, Ser110, Thr113) [M + 3H] ^3+^ at *m/z* 930.089 with a disialylated core 1 type glycan attached

**Figure 4:**
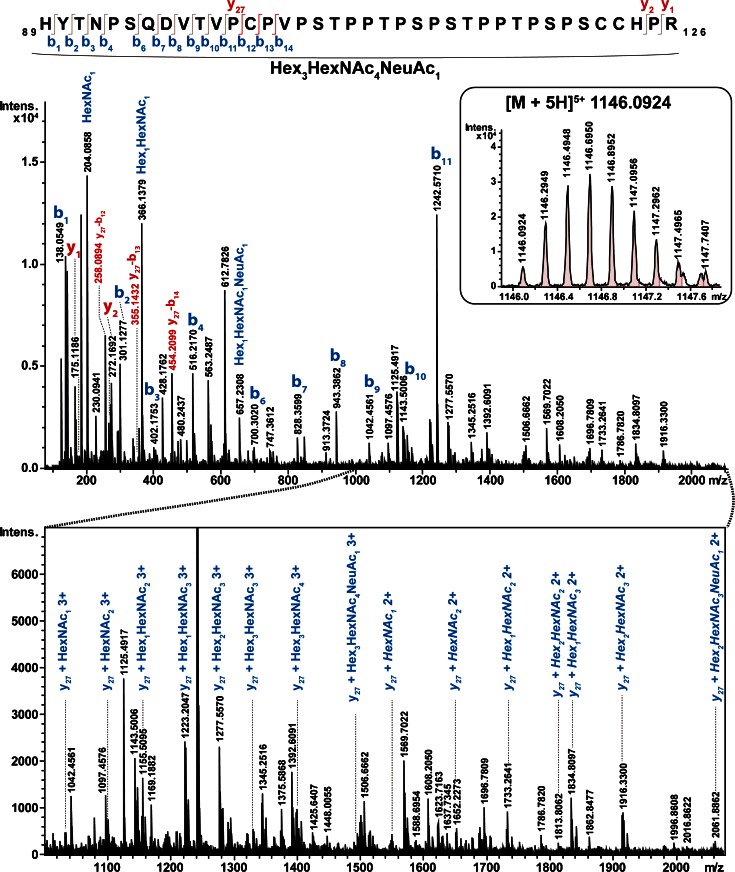
Product-ion spectra of a manually identified IgA1 *O*-glycopeptide using the optimized Q-TOF collision energy stepping dissociation method. The glycopeptide [M + 5H]^5+^ 1146.0924 with 89His-Arg126 has several occupied *O*-glycosylation sites with a total composition of Hex_3_HexNAc_4_NeuAc. The glycopeptide could not be identified automatically because the peptide backbone also fragmented into b_11_ and y_27_ + glycan fragments. Diagnostic b-ion fragments of the y_27_ initial fragment are highlighted in red. Note the specific, proline-rich nature of the peptide sequence, resulting in fragments with larger sequence gaps

The 10 glycoproteins investigated here have been previously reported to contain 33 N- and 18 O-glycosylation sites (Table 2). In silico digestion with trypsin (not taking missed cleavages into account) predicted 30 N-glycopeptides and five O-glycopeptides resulting from these glycoproteins. The individual tryptic digests were subjected to nanoRP-LC-ESI-Q-TOF tandem MS using the optimized method and without any further glycopeptide enrichment. Analysis of the obtained data with ProteinScape 4.0 revealed peptide sequences and glycan compositions of 21 tryptic N- and two O-glycopeptides (Table 2). The obtained spectra contained both features, B- and Y-ions resulting from the glycan fragmentation of lower-energy CID as well as b- and y-ions of the peptide portion attributable to the enhanced energy (Figure 3). ProteinScape also correctly classified seven additional spectra as glycopeptides, which could, however, not be positively associated with any protein by the software algorithm. Manual inspection of the spectra confirmed the initial glycopeptide classification, but also revealed the reason why they could not be automatically assigned. Most of these glyco-classified but unassigned spectra derived from glycopeptides with multiple sites of glycosylation. This holds true for the tryptic IgA1-peptide His89-Arg126, which can contain up to five O-glycosylation sites (Ser105, Ser111, Ser113, Ser119, Ser121). Additionally, this glycopeptide also shows a prominent fragmentation of the peptide backbone (b_11_, y_27_ + glycan) with retention of (parts of the) glycan moieties (Figure 4, Table 2), which prohibited the algorithm from determining a correct peptide mass of this highly complex glycopeptide.

IgD is the second glycoprotein in this panel for which up to seven sites of O-glycosylation have been reported earlier (Ser109, Ser110, Thr113, Thr126, Thr127, Thr131, and Thr132) [31, 32]. Similar to the IgA1 hinge-region glycopeptide (Figure 4), the detected tryptic glycopeptide Ala106-Arg137 (containing two missed cleavages) did not exhibit a fragmentation pattern, which resulted in a “peptide-only” or “peptide + HexNAc” fragment; instead a “peptide + Hex_2_HexNAc_3_NeuAc” was the smallest Y-type ion detected (Supplementary Figure 5). Thus, it was impossible for the ProteinScape algorithm to determine the right peptide mass value needed for automated glycopeptide assignment of this multiply glycosylated peptide. It is likely that an automated assignment of such complex product-ion spectra by currently available software tools will deliver similar results, though this was not further evaluated and tested in the course of this study. The remaining five glycosylation sites that were classified as glycopeptides but not identified by the software showed low-intensity peptide b- and y-ions and thus could not be assigned automatically.

The intrinsic nature of the residual, as yet undetected, glycopeptides from this sample set was most likely responsible for why they evaded identification or were not detected at all. The predicted tryptic glycopeptides from IgE (Asn17-Lys80 [N-glycan at N21 and N49]) and Ile116-Lys183 (N-glycan at N146), from bovine fetuin (Val246-Arg306 [O-glycans at Ser271, Thr280, Ser282, and Ser296]) and from IgA2 (Val8-Arg51 [N-glycan at N47]) are all large glycopeptides with a length of more than 40 amino acids. Under the applied analysis conditions these compounds are possibly too hydrophobic and therefore likely to be retained irreversibly on the C18 stationary phase.

In addition, multiply glycosylated peptides such as IgE Asn17-Lys80, IgM Thr269-Arg300, secretory component Ala82-Arg107, and fetuin Val246-Arg306 will also carry various charges and exhibit a higher glyco-heterogeneity compared with glycopeptides with a single site of glycosylation. These factors will additionally contribute to expected overall lower signal intensities, which also makes it less likely that these signals will be selected for tandem MS experiments under data-dependent selection criteria. As demonstrated earlier, the use of alternative and/or multiple proteases resulting in smaller, less heterogeneous sets of distinguished glycopeptides represents one opportunity to cover these sites [25, 33–35].

In the course of this study, no glycopeptides or peptides covering N327 from IgA2 could be identified, which might be explained by incomplete site occupation or lower ionization efficiency of the glycopeptide. Potential alterations on the C-terminus of the IgA2 used in this study could also result in different peptide backbones that evaded detection/identification. In contradiction to other studies claiming N92 in IgA2 to be quantitatively N-glycosylated despite the presence of a Proline within the N-glycosylation sequon (^89^HYTNPSQDVTVPCPVPPPPPCCHPR^113^) [36], we were not able to detect the respective site being N-glycosylated at all. Nevertheless, the non-glycosylated tryptic peptide could clearly be detected and identified (Supplementary Figure 6), which is also in line with previous in-depth glycoproteomic data on secretory IgA [37]. Even manual inspection for traces of glycosylation on this site did not, at least in the sample set analyzed, result in any detectable form of glycosylation on N92.

The optimized collision energy stepping CID conditions reported here were found to deliver solid glycan composition as well as peptide sequence information for precursor ions distributed over a wide *m/z* range. Compared with alternative fragmentation techniques such as ETD, we found collision energy stepping CID to be more robust in delivering useful data within a data-dependent LC-ESI tandem MS experiment, in particular if precursors with high *m/z* values were selected. Nevertheless, collision energy stepping CID also efficiently fragmented the linkage between the oligosaccharide and the peptide, making it practically impossible to determine sites of glycosylation by this approach. Though this is of less significance for N-glycosylated peptides because of the well preserved glycosylation sequon, it poses certain drawbacks for site determination of post-translational modifications where the specific sites cannot be reliably predicted from the protein sequence (e.g., O-glycans or phosphorylation). However, collision energy stepping CID still provides a valuable first data set on peptide sequence and glycan composition even for multiply O-glycosylated peptides (Figure 4 and Supplementary Figure 5). Based on such results, alternative and targeted approaches can subsequently be applied where both sample preparation and acquisition parameters can be optimized to gain site attachment information using ETD [38, 39].

The glycoforms reported in this study on the respective sites of glycosylation represent only glycoforms that were selected and confirmed by product-ion spectra (Table 2); thus, the data does not represent the full variety of glycosylation present on specific sites. Several additional glycoforms were obvious from the MS1 data but are not reported since identification on MS-level and overall site-specific glycosylation heterogeneity were not the scope of this study. It should be noted that all identified glycopeptides were in line with previously published literature (see references in Table 2) and with currently accepted, repeatedly verified general knowledge on protein N-glycosylation.

## Conclusions

Overall, the optimization of CID energy parameters for Q-TOF instruments using synthetic N-glycopeptides yielded improved fragmentation for both the glycan and the peptide moiety. To obtain optimal fragmentation data, peptide moieties required generally higher energies. Twice the energy (compared with the glycan portion) was applied for 80% of the fragmentation time to obtain sufficient peptide bond cleavages. Under the conditions applied in this study, the most informative glycopeptide spectra were obtained when collision energies of 55 eV at 700  *m/z* to 124 eV at 1800 * m/z* were applied in a *m/z* dependent manner.

The applied fragmentation conditions are well suited to a broad range of glycopeptides. This is exemplified by data showing that the positioning of the glycan moiety on the peptide and/or slight sequence differences only lead to a small change in optimum collision energy for the peptide moiety. Furthermore, the peptide portion did not influence the optimal fragmentation conditions for the glycan part of the glycopeptides, at least for the ones analyzed in this study.

Glycopeptide fragmentation aspects on Q-TOF instruments has been the subject of investigation previously [19–21]. One of the earliest reports by Jebanathirajah et al. described glycan and peptide specific fragmentation aspects using synthetic N- and O-glycopeptides carrying a disaccharide [20]. The non-tryptic nature of these compounds, however, is likely to affect the fragmentation compared with tryptic glycopeptides. Recently, Kolli and Dodds reported a systematic in-depth investigation of enriched, tryptic glycopeptides from bovine ribonuclease B and *Erythrina cristagalli* lectin [19]. In this excellent study that evaluated glycan as well as peptide fragmentation aspects, both investigated glycopeptides carried neutral N-glycans with a maximum of seven monosaccharide residues. Other reports mainly focused on the fragmentation characteristics of the glycan part only, without further dissociation of the peptide backbone [21]. The data presented here is, to the best of our knowledge, the first to systematically optimize fragmentation conditions of tryptic glycopeptides carrying complex biantennary, disialylated N-glycans and validate these optimized fragmentation parameters by performing a glycoproteomic investigation of the entire panel of human immunoglobulins (IgA, sIgA, IgD, IgE, IgG1, IgG2, and IgM) and bovine fetuin. Using the optimized MS parameters, most of the expected glycopeptides reported in literature could be identified. Peptide sequence as well as glycan composition data were obtained on a representative range of glycopeptides carrying N- as well as O-glycans.

Our results also highlight how bioinformatic tools strongly benefit from data acquired under optimized acquisition parameters. Software-assisted data analysis (ProteinScape 4.0) not only successfully identified *N*- and *O*-glycopeptides but also elucidated several glycoforms and made putative structure suggestions. The optimized methodology for glycopeptide analyses presented here using Q-TOF instruments and the associated computational tools will prove to be particularly useful in the fields of glycoproteomics research as well as biopharmaceutical development and quality control applications. The major challenges for glycopeptide characterization in complex biological sample matrices remain their lower ionization efficiency [12] and efficient and specific enrichment.

## Electronic supplementary material

ESM 1 (PDF 3.03 mb)
