# Supplementary material for: The Art of Destruction: Optimizing Collision Energies in Quadrupole-Time of Flight (Q-TOF) Instruments for Glycopeptide-Based Glycoproteomics
Source: J Am Soc Mass Spectrom. 2016 Jan 4;27:507–19. doi: 10.1007/s13361-015-1308-6 (PMC4756043; doi:10.1007/s13361-015-1308-6)
Supplement: Supplementary file 1 — (PDF 3.03 mb) [file 13361_2015_1308_MOESM1_ESM.pdf]

Supplementary Material to:

The art of destruction: Optimizing collision energies in quadrupole-time of flight (Q-TOF) instruments for glycopeptide based glycoproteomics

Hannes Hinneburg[1,2], Kathrin Stavenhagen[3], Ulrike Schweiger-Hufnagel[4], Stuart Pengelley[4], Wolfgang Jabs[4], Peter H. Seeberger[1,2], Daniel Varón Silva[1], Manfred Wuhrer[3,5], Daniel Kolarich[1]*

1 Department of Biomolecular Systems, Max Planck Institute of Colloids and Interfaces, 14424 Potsdam, Germany

2 Institute of Chemistry and Biochemistry, Freie Universität Berlin, Arnimallee 22, 14195 Berlin, Germany

3 Division of BioAnalytical Chemistry, VU University Amsterdam, Amsterdam, The Netherlands

4 Bruker Daltonik GmbH, Bremen, Germany

5 Center for Proteomics and Metabolomics, Leiden University Medical Center, Leiden, The Netherlands

* Address reprint requests to: Dr. Daniel Kolarich

Department of Biomolecular Systems, Max Planck Institute of Colloids and Interfaces, 14424 Potsdam, Germany

Telephone: +49-30-838 59306; Fax: +49-30-838 459306

Email: daniel.kolarich@mpikg.mpg.de

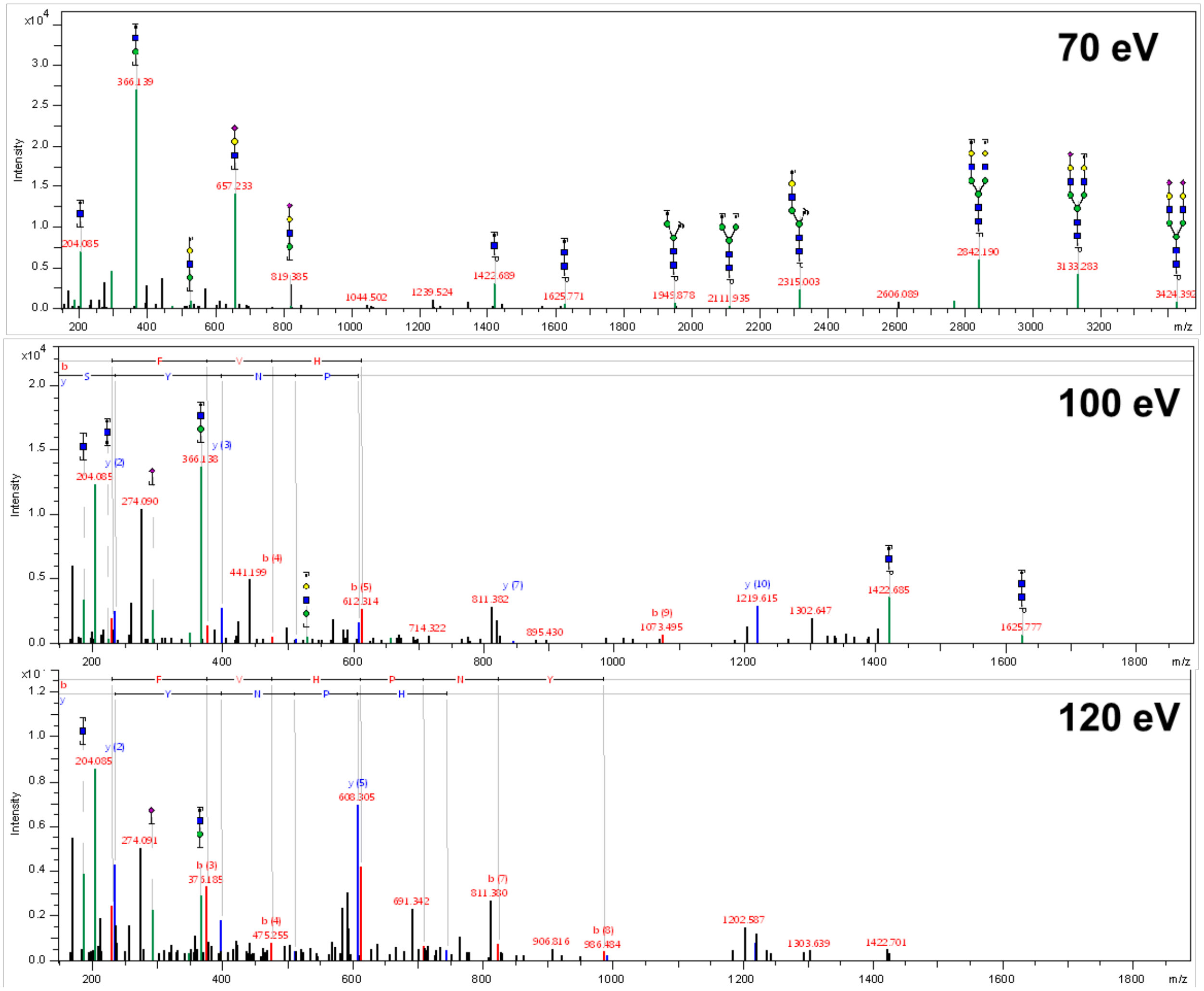

Supplementary Figure 1: Fragment spectra of GP-M under different collision energies. Fragment spectra obtained for GP-M ($[M + 2H]^{2+}$) for collision energies of 70 eV, 100 eV and 120 eV.

Human colon preparations (SDS PAGE; HILIC)

Membrane protein preparation of colon tissue was performed similar as described by Lee et al. [1]. In detail, the tissue pieces (~ 0.25 cm^2) were mixed with 2 mL of lysis buffer (pH 7.4) containing 50 mM Tris-HCl, 100 mM NaCl, 1 mM EDTA and protease inhibitor cocktail (Roche Diagnostics GmbH, Mannheim, Germany, 1 tablet per 50 mL) and incubated for 1 h on ice. After incubation another 3 mL of dilution buffer (20 mM Tris-HCl, 0.1 M NaCl) were added and the tissue was homogenized on ice using a Polytron for 15 seconds (7 cycles). The homogenate was centrifuged at 2,000 g for 20 min at 4°C to remove any debris and nuclei. The supernatant containing the proteins was further processed by ultracentrifugation at 120,000 g for 90 min at 4°C to sediment the proteins. The pellet was dried in a vacuum concentrator and stored in the freezer for further use. 20 µg of protein (pellet) were dissolved in 40% glycerin, 0.25 mM Tris-HCL pH 6.8, 0.015 % bromophenol blue (w/v), 4% SDS (w/v), 50 mM dithiothreitol and incubated at 96°C for 5 min. Then iodoacetamide was added to a final concentration of 50 mM and samples were incubated at room temperature for 30 min in darkness. Samples were then used for SDS-PAGE.

For electrophoresis runs a consort EV265 power supply (Consort bvba, Turnhout, Belgium) and a Mini-PROTEAN® Tetra Cell (Biorad, Munich, Germany) as well as self-cast polyacrylamide gels (0.75 mm thick) and Laemmli [2] system was used. Stacking gels (pH 6.8) consisted of 5% polyacrylamide, separation gels (pH 8.8) of 10%. The acrylamide/bis ratio in both gels was 29:1. The running buffer contained 25 mM Tris, 192 mM glycine, 0.1% SDS (w/v), pH 8.3. Electrophoresis was carried out with 160 V for 75 min. As marker Precision Plus Protein™ Dual Color Standard (Biorad, Munich, Germany) was used.

Gel (Supplementary Figure 2) was stained for 5 min with 50% (v/v) aqueous methanol containing 0.25% (w/v) Coomassie brilliant blue R-250 (Serva Electrophoresis GmbH, Heidelberg, Germany); and 7% (v/v) acetic acid. Excess dye was removed by destaining with 50% (v/v) methanol containing 10% (v/v) acetic acid for at least 3 h. Bands (box Supplementary Figure 2) were excised and in-gel trypsin digested [3]. The extracted peptides were pooled (six patients), dried in a vacuum concentrator and stored at -20°C until further use.

Extracted (glyco-)peptides were solved in 50 µL 80% ACN, 1% TFA and used for HILIC enrichment with 8 mg PolyHYDROXYETHYL A, (12-µm, 100-Å) (PolyLC Inc., Columbia; USA) packed onto an Empore™ C8 membrane plug (3M Deutschland GmbH, Neuss, Germany) in a 10 µL Eppendorf tip (Eppendorf AG, Hamburg, Germany) using a 5417 R Eppendorf centrifuge (Eppendorf AG, Hamburg, Germany). Precondition was with 3x 50 µL methanol and 50 µL

water followed by equilibration with 3x 50 µL 80% ACN, 1%TFA. After loading, column was washed 4x 50 µL 80% ACN, 1%TFA before bound compounds were eluted with 50 µL 0.1% TFA, 50 µL 25 mM ammonium bicarbonate and 50 µL 50% ACN. Fractions were combined, dried and dissolved in water or 2% ACN for MS analysis.

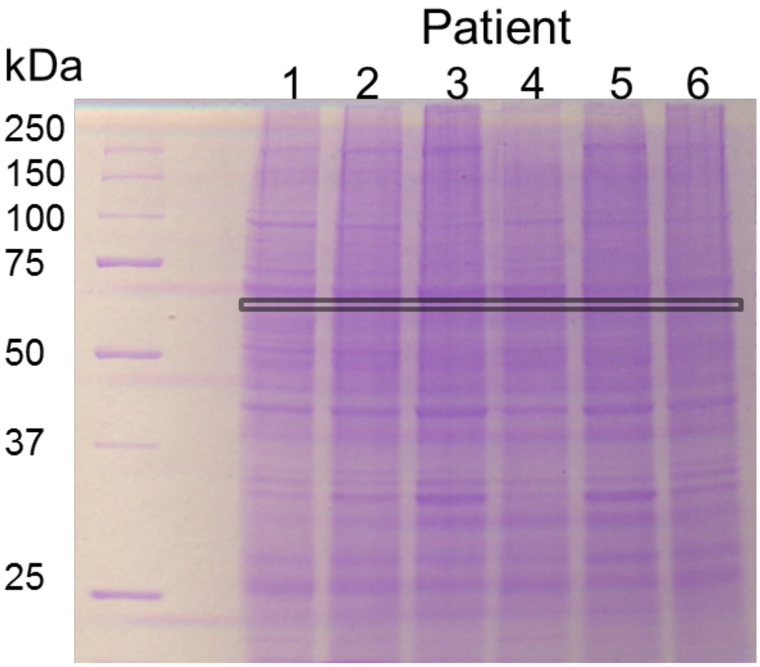

Supplementary Figure 2: SDS-PAGE separated human colon samples from patients with ulcerative colitis. Box indicates bands which were used for HILIC enrichment.

Supplementary Table 1: Glycopeptides analyses from human colon samples for evaluation of optimized collision energies obtained in experiments with synthetic glycopeptides.

m/z	**z**	**$[M+H]^+$**	**Peptide sequence**	**ProteinID**	**Composition (Hex, HexNAc, NeuAc, dHex)**
685.682	5	3424.383	EVFVHPNYSK	PROC_HUMAN	5/4/2/0
856.851	4	3424.383	EVFVHPNYSK	PROC_HUMAN	5/4/2/0
869.395	3	2606.170	K.TPLTANITK.S	IGHA2_HUMAN	3/5/0/1
1142.133	3	3424.383	EVFVHPNYSK	PROC_HUMAN	5/4/2/0
1200.849	2	3600.533	R.TILVDNNTWNNTHISR.V	STT3A_HUMAN	8/2/0/0
1254.867	3	3762.586	R.TILVDNNTWNNTHISR.V	STT3A_HUMAN	9/2/0/0
1230.560	2	2460.112	K.TPLTANITK.S	IGHA2_HUMAN	3/5/0/0
1303.589	2	2606.170	K.TPLTANITK.S	IGHA2_HUMAN	3/5/0/1
1384.615	2	2768.223	K.TPLTANITK.S	IGHA2_HUMAN	4/5/0/1
1395.621	2	2790.234	R.NMSNQLGLLAVNQR.F	PERM_HUMAN	5/2/0/0
1428.623	2	2856.239	K.TPLTANITK.S	IGHA2_HUMAN	4/4/1/1
1476.647	2	2952.287	R.NMSNQLGLLAVNQR.F	PERM_HUMAN	6/2/0/0
1530.163	2	3059.318	K.TPLTANITK.S	IGHA2_HUMAN	4/5/1/1
1648.648	2	3296.288	K.EVFVHPNYS.K	PROC_HUMAN	5/4/2/0
1712.695	2	3424.38	EVFVHPNYSK	PROC_HUMAN	5/4/2/0
1728.173	2	3455.338	LGNWSAMPSCK	APOH_HUMAN	5/4/2/0

Hex: Hexose; NexNAc: N-Acetylhexosamine; NeuAc: N-acetylneuraminic acid; dHex: deoxy-hexose.

Human Ig preparations

Approximately 5 µg **IgA** (plasma), **sIgA** (human colostrum), **IgD** (plasma), **IgE** (myeloma plasma, lambda), **IgG1** (myeloma plasma, kappa), **IgG2** (myeloma plasma, kappa) and **IgM** (plasma) were dissolved and reduced and alkylated as described above. Electrophoresis run, staining and in-gel tryptic digest was done as described above. Excised bands are indicated in Supplementary Figure 3.

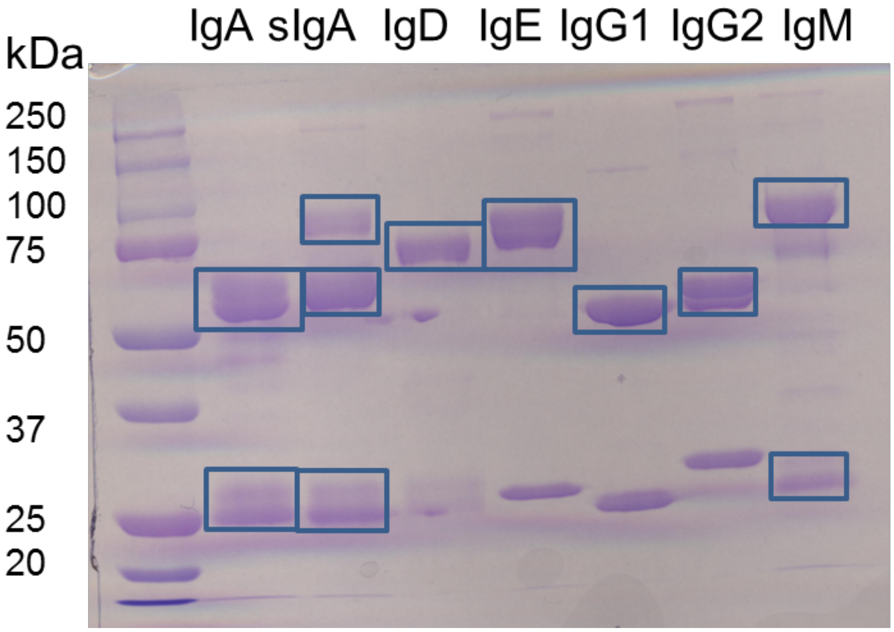

Supplementary Figure 3: SDS-PAGE separated immunoglobulins. Boxes indicate bands cut for tryptic digestion.

Software assisted glycopeptide data analysis - Parameter settings

For glycopeptide identification the two data sets were classified with two peptide mass off sets (H+ and HexNAc + H+) before the glycan and protein search was done and results were combined. The parameters for the integrated protein data base searches (mascot used as search engine) as well as the parameters for the glycan searches can be seen in Supplementary Figure 4. Note that the mascot scores for acceptance (protein, peptide) were set to relatively low values which is necessary due to the search engine scoring algorithm which calculates low values if unassigned peaks with relative high intensities (glycan fragments!) occur in the MS/MS peptide spectrum.

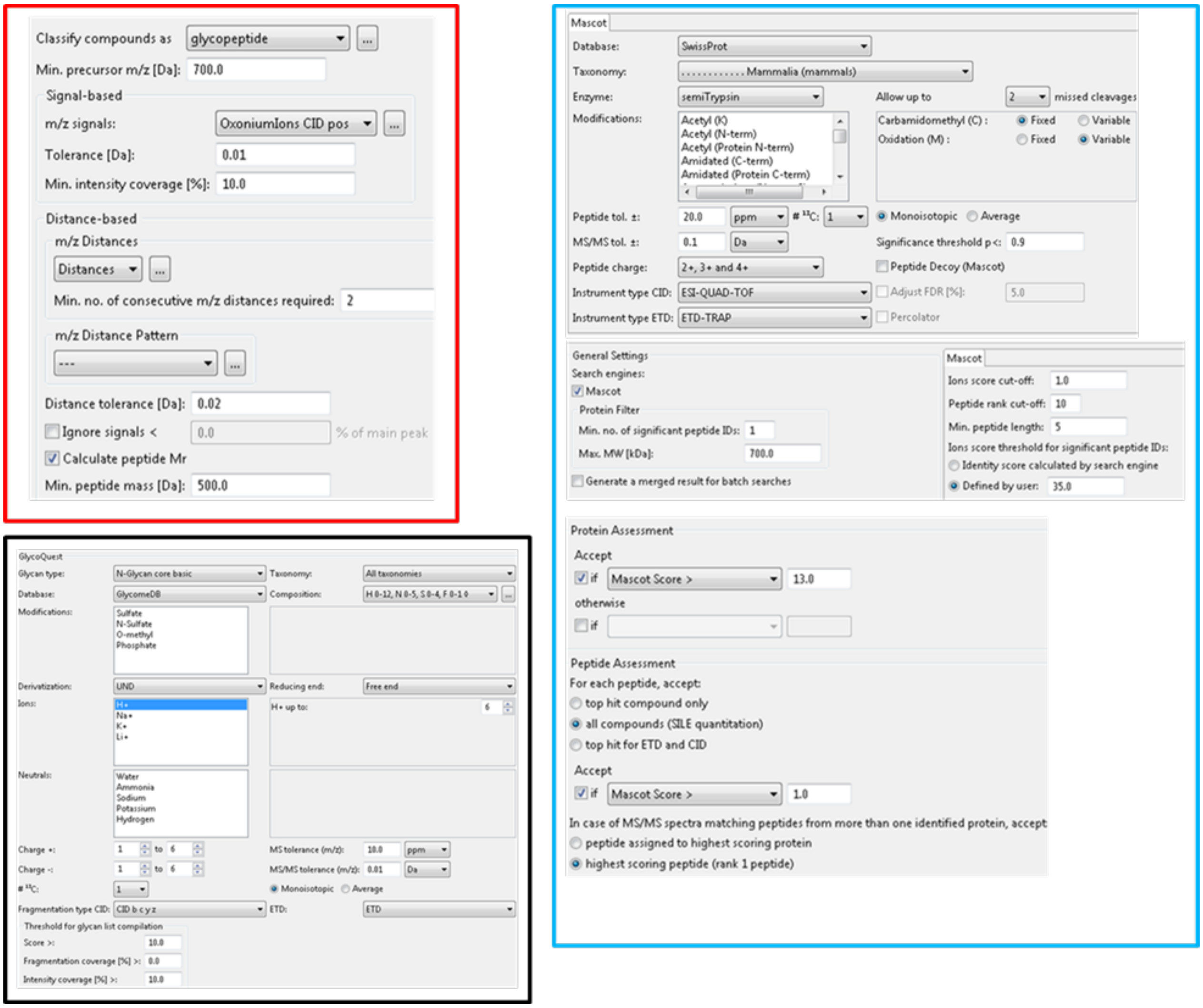

Supplementary Figure 4: ProteinScape search parameters. Parameter settings for classification (red box), protein identifications (blue box) and glycan searches (black box) using ProteinScape 4.0 for automated glycopeptide recognition.

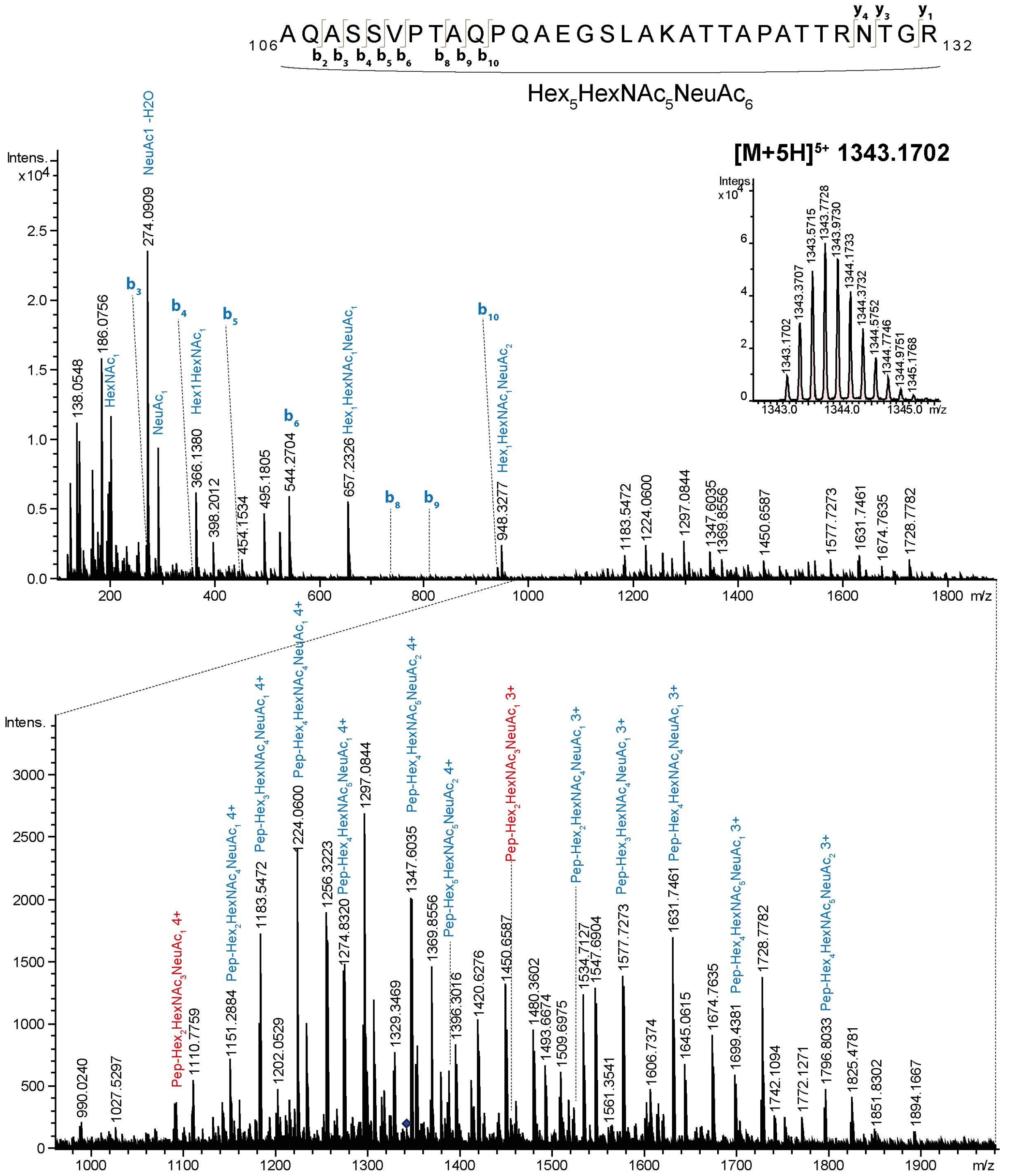

Supplementary Figure 5: MS/MS fragmentation spectra of a manually identified IgD O-glycopeptide. The glycopeptide [M + 5H]$^{5+}$ 1343.1702 with 106Ala-Arg132 has several occupied O-glycosylation sites with a total composition of $Hex_5HexNAc_5NeuAc_6$. The glycopeptide could not be identified automatically because the fragmentation of the Y-ion series of the glycan part was not complete down to the last attached HexNAc or the peptide backbone. Instead the smallest Y-ion still contains $Hex_2HexNAc_3NeuAc$ (indicated in red).

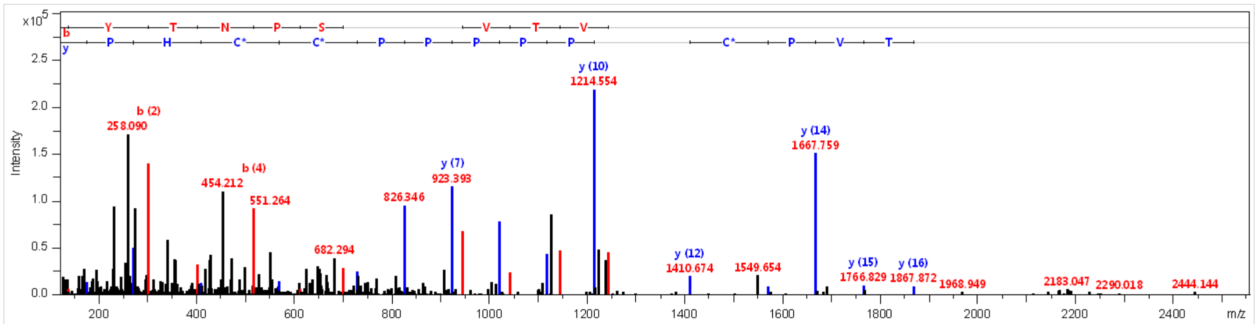

Supplementary Figure 6: MS/MS fragmentation spectrum of the tryptic peptide 89HYTNPSQDVTVPCPVPPPPPCCHPR113 from IgA2. This peptide was just detected in its unglycosylated form, and no indications for any traces of N- or O-glycosylation on this peptide could be detected. This is in line with current knowledge about the specificity of the oligosaccharyltransferase.

References

1. Lee, A., Kolarich, D., Haynes, P.A., Jensen, P.H., Baker, M.S., Packer, N.H.: Rat liver membrane glycoproteome: enrichment by phase partitioning and glycoprotein capture. Journal of proteome research. **8**, 770-781 (2009)
2. Laemmli, U.K.: Cleavage of structural proteins during the assembly of the head of bacteriophage T4. Nature. **227**, 680-685 (1970)
3. Kolarich, D., Jensen, P.H., Altmann, F., Packer, N.H.: Determination of site-specific glycan heterogeneity on glycoproteins. Nat Protoc. **7**, 1285-1298 (2012)
